# Severe anaphylaxis during sentinel lymph node biopsy (SLNB)

**DOI:** 10.1186/2045-7022-4-S3-P5

**Published:** 2014-07-18

**Authors:** Andreia Ferrão Vasconcelos Pinela, Cintia Cruz, Elza Tomaz, Filipe Inacio

**Affiliations:** 1 Hospital da Misericórdia de Évora, Department of Immuno-allergology, Portugal; 2 Centro Hospitalar Setúbal, Department of Immuno-allergology, Portugal

## 

The aim of this study was to present two cases of severe anaphylaxis during sentinel lymph node biopsy (SLNB). Case 1: A 64-year-old-woman had two months earlier been diagnosed with breast ductal invasive carcinoma by first local excision. She was taken for further surgery with SLNB. At induction she received midazolam, fentanyl, propofol, atracurium, cefazolin and paracetamol. She was given a subcutaneous injection of the dye Patent Blue V (PBV) and shortly afterward she developed a blue rash over her abdomen and her blood pressure dropped precipitously. She was treated successfully with intravenous adrenaline and fluids. Six weeks later, a skin prick test (SPT) and intradermal test (ID) to all used drugs, latex and B-lactamics were performed. SPT and ID to PBV were positive with 3mm and 6.5m in weal diameter. ID test to cefazolin was also positive (6.5mm). All others were negative. Case 2: A 49-year-old woman required local excision of a breast lump with SLNB. She received midazolam, fentanyl, cefazolin and atracurium. PBV was then injected and 15 min later, she developed generalized erythema and severe hypotension and bradycardia. She required, intravenous adrenaline and amines infusion postanaesthetic. SPT and ID to all used drugs, latex and B-lactamics were performed. SPT to PBV diluition 1:100 and 1:10 were positive with 3mm and 8mm in weal diameter. The ID testing of atracurio in major concentration of 1:1000 revealed a 7 mm increase in the weal. All others were negative.

## Discussion

In case 1 the PBV positive test, the blue rash, and the positive test to cefazolin with negative B-lactamic test indicate a type I allergy towards PBV as a more probable cause of the perioperative anaphylaxis and a selective sensibilization to cefalosporins. In case 2 the positive reaction to SPT to PBV suggest the diagnosis of patent blue anaphylaxis. The positive ID test to atracurium in major concentration is probably indicative of an irritant. The etiological diagnosis in perioperative anaphylaxis remains complex and often difficult to accomplish.

